# Regional Inequalities in Lung Cancer Mortality in Belgium at the Beginning of the 21st Century: The Contribution of Individual and Area-Level Socioeconomic Status and Industrial Exposure

**DOI:** 10.1371/journal.pone.0147099

**Published:** 2016-01-13

**Authors:** Paulien Hagedoorn, Hadewijch Vandenheede, Didier Willaert, Katrien Vanthomme, Sylvie Gadeyne

**Affiliations:** Interface Demography, Department of Sociology, Faculty of Economic and Social Sciences and Solvay Business School, Vrije Universiteit Brussel, Brussels, Belgium; Tsinghua University, CHINA

## Abstract

Being a highly industrialized country with one of the highest male lung cancer mortality rates in Europe, Belgium is an interesting study area for lung cancer research. This study investigates geographical patterns in lung cancer mortality in Belgium. More specifically it probes into the contribution of individual as well as area-level characteristics to (sub-district patterns in) lung cancer mortality. Data from the 2001 census linked to register data from 2001–2011 are used, selecting all Belgian inhabitants aged 65+ at time of the census. Individual characteristics include education, housing status and home ownership. Urbanicity, unemployment rate, the percentage employed in mining and the percentage employed in other high-risk industries are included as sub-district characteristics. Regional variation in lung cancer mortality at sub-district level is estimated using directly age-standardized mortality rates. The association between lung cancer mortality and individual and area characteristics, and their impact on the variation of sub-district level is estimated using multilevel Poisson models. Significant sub-district variations in lung cancer mortality are observed. Individual characteristics explain a small share of this variation, while a large share is explained by sub-district characteristics. Individuals with a low socioeconomic status experience a higher lung cancer mortality risk. Among women, an association with lung cancer mortality is found for the sub-district characteristics urbanicity and unemployment rate, while for men lung cancer mortality was associated with the percentage employed in mining. Not just individual characteristics, but also area characteristics are thus important determinants of (regional differences in) lung cancer mortality.

## Introduction

Lung cancer is one of the most common cancers worldwide. In 2002 an estimated 1.18 million lung cancer deaths were recorded, accounting for 18% of cancer deaths and 2% of total mortality [1]. In Belgium, lung cancer mortality is relatively low among women, but steadily expanding and expected to increase in the future [2]. Belgian men have the highest lung cancer mortality rates of Western Europe [3]. Belgium is a densely populated area with one of Europe’s highest levels of air pollution [4] and a high concentration of industry posing a risk to air, water and soil quality [5]. This high-risk setting makes Belgium an interesting study area for further research into the lung cancer epidemic.

Moving beyond the overall country-level pattern through exploring geographical differences within a country gives valuable insights into at risk areas and populations and can identify possible risk factors for increased lung cancer mortality. A recent study identified regional variation in lung cancer incidence in Belgium [6], yet little is known about regional variation in lung cancer mortality and its underlying determinants. This study therefore aims to investigate geographical patterns in lung cancer mortality in Belgium on a sub-district level and wishes to probe into the contribution of individual as well as area-level characteristics to these geographical patterns. The data consist of a unique and exhaustive dataset based on individually linked census mortality follow-up data for the total de jure population of Belgium. This enables us to study the impact of individual and area-level socioeconomic status (SES) and industrial exposure on lung cancer mortality, both at the individual and sub-district level.

As for individual socioeconomic characteristics, multiple studies have found a strong socioeconomic gradient in lung cancer incidence and mortality with increased risks among individuals with a lower SES [7,8]. Additionally, at an individual level, several occupational carcinogenic agents have been associated with lung cancer, including asbestos, arsenic, beryllium, cadmium, chromium, nickel, silica and diesel engine exhaust [9,10]. Differences in lifestyle, especially smoking, and occupational exposure are possible pathways through which individual characteristics might impact lung cancer mortality [8].

At the regional level, there are three main factors resulting in regional mortality variations. The first is the compositional effect of individual characteristics, whereby individuals with certain characteristics are clustered spatially [11]. Secondly, the contextual socioeconomic characteristics of the neighborhood and living environment also appear to influence health and all-cause mortality, independently from individual SES [12,13]. This effect of area-SES has also been found for cancer mortality [14–16] and lung cancer mortality [14,16–18], although some studies found little or no effect of area deprivation on lung cancer mortality [15,19]. Thirdly, physical environmental factors could result in regional inequalities in lung cancer mortality. Individuals living close to industrial installations, especially around metal industries, cement plants and shipyards, generally experience increased lung cancer risks [20]. Residents of coal-mining areas were found to have increased levels of lung cancer mortality as well [21]. On the other hand, some studies did not find a clear association between industrial pollution and lung cancer [22,23]. Within Belgium, exposure to cadmium from local zinc smelters has been associated with increased levels of lung cancer incidence among residents of Northeast Belgium [24].

There have been ample studies looking into the effects of individual SES [7,8] and environmental pollution on lung cancer mortality [20,24,25], but relatively little is known about the role of area SES [17]. Few studies investigated all three factors at the same time in relation to lung cancer mortality, even though studying individual- and area-level characteristics can provide valuable information about disease etiology and at-risk areas and populations. Our exhaustive dataset allows us to study the joint effects of individual and area SES and industrial exposure on lung cancer mortality in Belgium. A first aim is to identify sub-district level variations in lung cancer mortality; a second aim is to provide explanations for observed patterns by quantifying the contributions of individual SES, urbanicity, sub-district level unemployment rate and the proportion employed in mining and in other high-risk industries.

## Material and Methods

### Data

The data consist of a linkage between the 2001 census and register data on survival status, emigration status and cause-of-death information for the period October 1, 2001 to December 31, 2011. The data cover the entire de jure population of Belgium and take all lung cancer deaths (defined by ICD-10 codes C33-C34) from 2001 to 2011 into account. As most lung cancer deaths occur at older ages, the study population is limited to men and women aged 65 years and older. The sample size consists of 1,742,356 persons (714,535 men and 1,027,821 women) and 36,099 lung cancer deaths (28,883 in men and 7,216 in women). This study was approved by the Belgian Commission for the Protection of Privacy. Individual record linkage between census and register data was conducted by Statistics Belgium, the government agency in charge of Belgian national statistics. The dataset was anonymized by Statistics Belgium before it was made available for research. All personal identifying information was thus removed from the dataset prior to analysis.

### Unit of analysis

**Fig 1:**
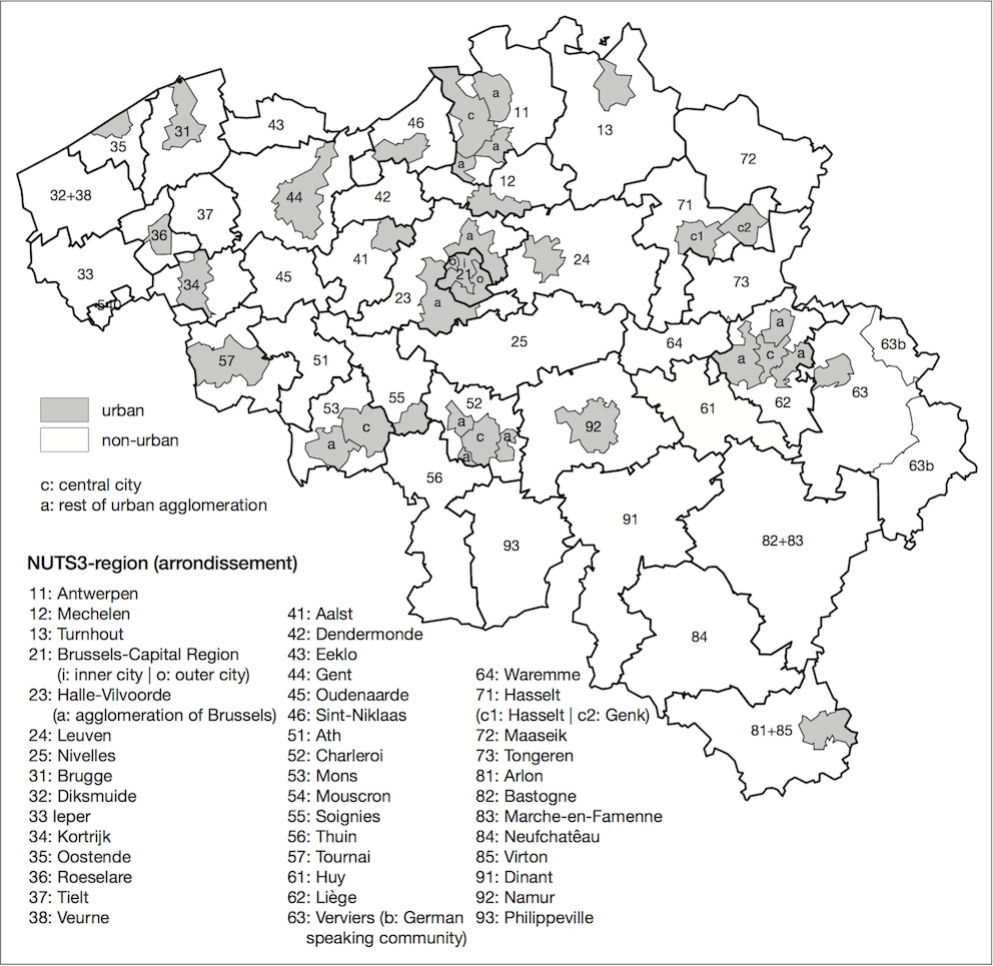
Belgian sub-districts. Cartography: Didier Willaert

The geographic unit of analysis used in this study is the sub-district, a manually constructed spatial unit to enable the distinction between urban and non-urban areas while ensuring geographical units large enough for robust analyses. The starting point is the administrative district (“arrondissement”) (N = 43), a spatial level between provinces and municipalities, corresponding to the Eurostat Nomenclature of Territorial Units for Statistics (NUTS) level 3. We identified urban areas using the classification of Belgian metropolitan areas by Luyten and Van Hecke (2007). Based on morphological and functional criteria (such as population density, land use, and commuter flows) they demarcated 18 urban agglomerations in Belgium [26]. If a district contains an urban agglomeration, we subdivided the district into an urban and a non-urban sub-district. As the district of Hasselt contains two urban agglomerations, a separate urban sub-district is constructed for each agglomeration. Large metropolitan areas (for example Antwerp and Liege) are further subdivided into two urban sub-districts, one containing the inner city, and one containing the surrounding agglomeration. Brussels is split into two urban sub-districts, one containing the “inner city” and the other the “outer city”. Districts that do not contain an urban agglomeration are not further subdivided and are classified as non-urban. There are three pairs of adjacent non-urban districts that have a relatively small number of inhabitants (Diksmuide and Veurne, Bastogne and Marche-en-Famenne, Arlon and Virton). These pairs are grouped together to ensure each spatial unit contains at least 50,000 inhabitants. In this way, a total of 68 regions are demarcated, of which 28 are classified as urban and 40 as non-urban (Fig 1). The number of lung cancer deaths and person years by sub-district varies from 18 deaths and 34,326 person years in women (Philippeville) to 1,064 deaths and 211,356 person years in men (central city of Antwerp).

#### Measurement of individual characteristics

There are several indicators for individual SES, including education, income, occupation, and housing status [27], although a combination of variables into one index can be used as well [28]. This study measures individual SES using information from the 2001 census on education, housing comfort and home ownership. Education captures knowledge and skills, income and housing comfort and home ownership capture material resources [27]. Each indicator thus measures a different aspect of SES, capturing SES more aptly [28].

As the majority of individuals aged 65+ were retired, employment status was not included. Education is based on the highest level of education attained and is categorized into four groups based on the International Standard Classification of Education (ISCED): (1) primary education or less (ISCED 0–1); (2) lower secondary education (ISCED 2); (3) upper and post-secondary education (ISCED 3–4); and (4) tertiary education (ISCED 5–6). Housing comfort is a weighted indicator based on the number of large repairs needed, living space, number of bedrooms and amenities. Housing comfort is classified into five categories: low quality; basic quality; good quality; good quality and spacious; and high quality and spacious [29]. Home ownership is a dummy variable indicating whether an individual is tenant or owner. Being married is shown to have a protective influence on cancer risk [30]. Marital status (married/non-married) is therefore included as a control variable together with age in years in 2001.

In total 473,067 individuals (27.2%) have a missing value on one of the individual SES variables. Lung cancer mortality rates for individuals with missing data on educational attainment and/or home ownership are slightly higher and lower, respectively, compared to the reference group (results not shown), while individuals with missing information on comfort level did not show significantly different mortality rates. However, including persons with missing values did not substantially alter the model outcomes, and they were therefore excluded. As a result, the final study population consists of 1,269,289 individuals (543,407 men and 725,882 women).

#### Measurement of sub-district level characteristics

Urbanicity, unemployment rate, proportion miners and proportion other high-risk industries are included as variables at sub-district level. Urbanicity is indicated by a dummy variable (urban/non-urban). Sub-districts belonging to an urban agglomeration as defined by Luyten and Van Hecke (2007) are classified as urban. Sub-districts outside an urban agglomeration are classified as non-urban. The unemployment rate is included as a proxy of area deprivation, and is calculated by the number of unemployed individuals as a percentage of the total labor force based on the 2001 census. The unemployment rate is expressed in quartiles; each quartile includes an approximately equal number of areas.

Environmental exposure to industrial pollution is estimated through the share of total industries that are considered high-risk industries per sub-district, measured by two variables: the percentage of the employed population employed in mining, and the percentage of the employed population employed in other high-risk industries. To incorporate the lag time between exposure and disease onset, both variables are based on the situation in 1981. The variables were constructed using a four-step procedure. First, the total population employed by industry is determined by sub-district. Second, manual workers in mining and in other high-risk industries are identified as working in either mining or high-risk industries respectively, whereas non-manual workers are not. Ahrens and Merletti [10] constructed a list of industries and occupations with a known risk of lung cancer, including mining; gas production; asbestos production; metal industries; ship, vehicle, and railroad manufacturing; construction workers and painters. Coal mining is the main mining industry in Belgium, however, coal is not confirmed as a carcinogenic by the IARC [31] nor is it included in the list by Ahrens and Merletti. Coal dust on the other hand, does contain several carcinogenic substances including zinc, arsenic and cadmium [32] and might expose local residents to environmental contamination through air or water [21]. Coal mining is therefore added to the mining types mentioned by Ahrens and Merletti (which include metal ore mining, and mining of certain non-metallic minerals). The remaining industries on their list are categorized as other high-risk industries. Third, the total number employed in mining, the total number employed in other high-risk industries and the total employed population are aggregated by sub-district. Fourth, the percentage employed in mining per district is calculated by dividing the total number of manual workers employed in mining by the employed population. The percentage employed in other high-risk industries per district is calculated by dividing the total number of manual workers in other high-risk industries by the employed population. Both variables are categorized in quartiles to aid interpretation.

### Methods

First, to get an overview of geographical patterns in lung cancer mortality in Belgium, the age-standardized mortality rate (ASMR), directly standardized to the 2001 Belgian population, was calculated by sub-district. The 95% confidence intervals for the ASMR were calculated based on a gamma distribution, which is more reliable when counts are small and variable [33]. Second, the association between lung cancer mortality and individual and sub-district characteristics, and their contribution to geographical mortality differences is estimated using multilevel Poisson models. As the hierarchical data structure and the non-independence of observations are aptly taken into account, multilevel models will result in more accurate estimates compared to single-level models [34]. Furthermore, multilevel models allow for the estimation of sub-district-level variation in lung cancer mortality and for the estimation of the extent to which individual and sub-district level characteristics can account for this variation [14]. A random intercept Poisson model is used including individuals at level 1 and sub-districts at level 2. The log of the person years is used as offset to account for differing exposure times. The model assumes that the effects of individual and sub-district level characteristics are similar across sub-districts, while lung cancer mortality at sub-district level (random intercept) is allowed to vary. The average relative deviation (ARD) for each model is obtained by post estimation[35], and is used to express the percentage deviation of the sub-district-variation from the total lung cancer mortality rate [19]. All analyses were conducted using Stata version 13.1 [36].

All models are conducted for men and women separately, and are restricted to people of 65 years and older. First, the null model is estimated. This model is adjusted for age only and serves as the baseline model. Additionally, three subsequent models were estimated, each controlled for age and marital status. Model 1 includes individual SES and is expanded stepwise by including urbanicity and unemployment rate (model 2), urbanicity and high-risk industry (percentage miners, and percentage other high-risk industries) (model 3). Model 4 includes all covariates (individual SES, urbanicity, unemployment rate and high-risk industry).

To test for the robustness of the findings, several supplementary analyses have been conducted. The models were repeated for the native Belgian population; for individuals residing in the same municipality in 1991 and 2001 (assuming a stable residence for over 10 years); and at municipal level. This did not substantially alter the results, nor when the models were repeated including missing values for individual SES, coded as a separate category. In addition, the models for men were repeated including individual occupation in 1981. This analysis could only be conducted for a subset of the male population (57%) who could be linked to the 1981 census and where information on occupation was available. Men employed as manual worker in other high-risk industries have a significantly higher lung cancer mortality risk. However, the inclusion of individual occupational exposure did not affect the outcomes for either individual SES or sub-district characteristics substantially. Finally, the models including the percentage employed in mining were repeated, further breaking down mining into coal mining and other mining types. Results of the supplementary analyses are available upon request.

## Results

### Regional variation in lung cancer mortality

**Fig 2:**
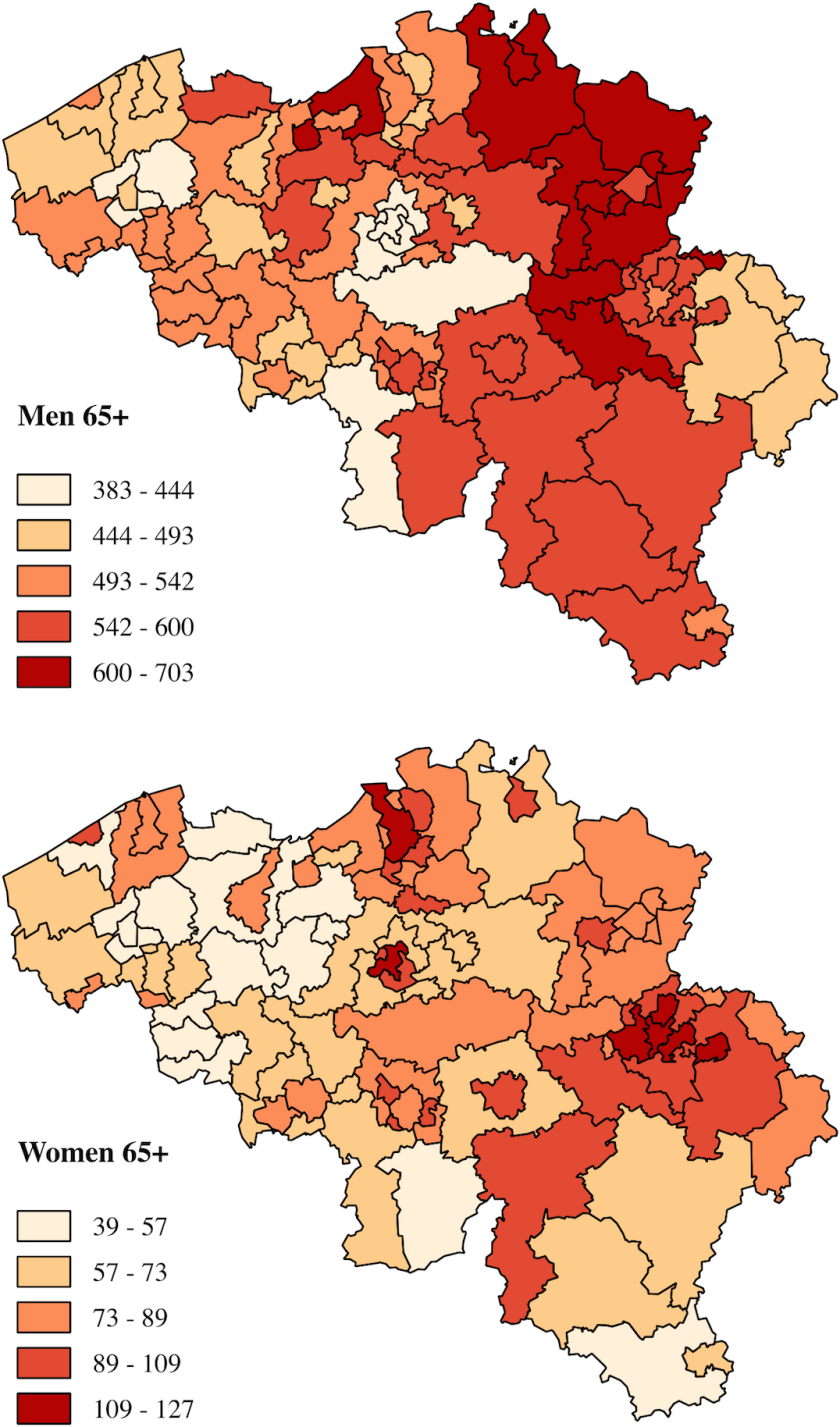
Directly standardized* lung cancer mortality rates for Belgium 2001–2011, men and women aged 65+. *Directly standardized to the 2001 Belgian population. Source: Belgian 2001 census linked to the National Register (2001–2011)

The geographical patterns for lung cancer mortality in Belgium for men and women aged 65+ are visualized in Fig 2. The maps show clear regional disparities in lung cancer mortality in Belgium, but also clear differences in the geographical pattern for men and women. Among men an east-west pattern is visible with high lung cancer mortality rates located in the east of Belgium, while among women high lung cancer mortality rates tend to be clustered in the cities. Brussels is an interesting case as the lung cancer mortality rate is among the lowest for men and among the highest for women. Table 1 shows the random variation in lung cancer mortality at sub-district level. The null model shows significant sub-district-level variation for both men and women. Geographical differences in lung cancer mortality are largest among women; their average relative deviation (ARD = 22%) is higher compared to that of men (ARD = 8.8%).

**Table 1:** Random-intercept variance in lung cancer mortality between sub-districts by model.

	Null model^a^	Model 1^b^	Model 2^c^	Model 3^d^	Model 4^e^
*Variance (standard error) *					
Men 65+	0.109 (0.012) *	0.109 (0.012) *	0.098 (0.012) *	0.081 (0.011) *	0.074 (0.010) *
Women 65+	0.248 (0.027) *	0.225 (0.026) *	0.149 (0.024) *	0.152 (0.023) *	0.130 (0.023) *
*Average Relative Deviation*					
Men 65+	8.8%	8.2%	7.6%	5.8%	5.2%
Women 65+	22.0%	18.5%	9.8%	10.7%	8.2%
*% Change from null model*					
Men 65+		6.9%	14.3%	33.8%	41.0%
Women 65+		15.7%	55.4%	51.2%	62.6%

* P<0.05

^a^Null model: adjusted for age only

^b^Model 1: includes individual SES, adjusted for age and marital status

^c^Model 2: includes individual SES, urbanicity and unemployment rate, adjusted for age and marital status

^d^Model 3: includes individual SES, urbanicity and environmental pollution, adjusted for age and marital status

^e^Model 4: includes individual SES, urbanicity and unemployment rate and industrial pollution, adjusted for age and marital status

### Association of individual SES with lung cancer mortality

Individual SES is negatively associated with lung cancer mortality, and individuals with a higher SES have a lower risk of lung cancer mortality (Table 2 and Table 3; model 1). Compared to low-educated men, highly educated men have a substantially lower lung cancer mortality risk (MRR = 0.55 [95%CI 0.53–0.58]). Highly educated women have a lower lung cancer mortality risk as well (MRR = 0.82 [95%CI 0.73–0.92]), however the lung cancer mortality among women with an upper- and post-secondary education does not differ from that of the low-educated women. Men living in good-quality housing experience lower levels of lung cancer mortality compared to men living in low-quality housing. Among women, housing comfort appears to have little association with lung cancer mortality. Ownership of a house on the other hand results in lower lung cancer mortality among women (MRR = 0.65 [95%CI 0.61–0.69]) compared to women who rent. A similar beneficial effect of home ownership on lung cancer mortality is observed for men (MRR = 0.76 [95%CI 0.74–0.79]). Marital status for men is not significantly associated with lung cancer mortality, while for women being married results in a significantly lower lung cancer mortality risk (MRR = 0.78 [95%CI 0.73–0.83]).

**Table 2:** Age-adjusted lung cancer mortality rate ratios (MRR) with 95% confidence intervals (CI) by individual and sub-district characteristics 2001–2011, men aged 65+.

	Model 1^a^		Model 2^b^		Model 3^c^		Model 4^d^	
	MRR	95%CI	MRR	95%CI	MRR	95%CI	MRR	95%CI
**Individual characteristics **								
*Educational level*								
Pre-primary/primary (ref.)	1.00	-	1.00	-	1.00	-	1.00	-
Lower secondary	0.84	(0.81–0.87)	0.84	(0.81–0.87)	0.84	(0.81–0.87)	0.84	(0.81–0.87)
Upper and post secondary	0.72	(0.69–0.76)	0.72	(0.69–0.76)	0.72	(0.69–0.76)	0.72	(0.69–0.76)
Tertiary	0.55	(0.53–0.58)	0.55	(0.53–0.58)	0.55	(0.53–0.58)	0.55	(0.53–0.58)
*Comfort level*								
Low quality (ref.)	1.00	-	1.00	-	1.00	-	1.00	-
Basic quality	1.02	(0.97–1.07)	1.02	(0.97–1.07)	1.02	(0.97–1.07)	1.02	(0.97–1.07)
Good quality	0.90	(0.86–0.94)	0.90	(0.86–0.94)	0.90	(0.86–0.94)	0.90	(0.86–0.94)
High quality	0.93	(0.89–0.98)	0.93	(0.89–0.98)	0.93	(0.89–0.98)	0.93	(0.88–0.98)
Very high quality	0.84	(0.79–0.89)	0.84	(0.78–0.89)	0.84	(0.79–0.89)	0.83	(0.78–0.89)
*Home ownership*								
Tenant (ref.)	1.00	-	1.00	-	1.00	-	1.00	-
Owner	0.76	(0.74–0.79)	0.76	(0.74–0.79)	0.76	(0.74–0.79)	0.76	(0.74–0.79)
*Marital status*								
Non-married (ref.)	1.00	-	1.00	-	1.00	-	1.00	-
Married	0.99	(0.96–1.02)	0.99	(0.96–1.02)	0.99	(0.96–1.02)	0.99	(0.96–1.02)
**Sub-district characteristics**								
*Urbanicity*								
Non-urban (ref.)			1.00	-	1.00	-	1.00	-
Urban			0.97	(0.91–1.04)	1.00	(0.95–1.06)	1.03	(0.97–1.10)
*Unemployment rate*								
Q1 (lowest) (ref.)			1.00	-			1.00	-
Q2			1.10	(1.01–1.20)			1.01	(0.93–1.09)
Q3			1.13	(1.05–1.23)			0.94	(0.85–1.03)
Q4 (highest)			1.06	(0.97–1.16)			0.89	(0.81–0.99)
*% Employed in mining*								
Q1 (lowest) (ref.)					1.00	-	1.00	-
Q2					1.09	(1.01–1.17)	1.08	(1.01–1.15)
Q3					1.13	(1.05–1.21)	1.19	(1.10–1.30)
Q4 (highest)					1.22	(1.13–1.31)	1.30	(1.18–1.43)
*% Employed in other high-risk industries*								
Q1 (lowest) (ref.)					1.00	-	1.00	-
Q2					0.94	(0.88–1.02)	0.94	(0.87–1.00)
Q3					0.97	(0.90–1.04)	0.97	(0.91–1.04)
Q4 (highest)					0.97	(0.90–1.04)	0.96	(0.89–1.03)

Ref. = Reference group

^a^Model 1: includes individual SES, adjusted for age and marital status

^b^Model 2: includes individual SES, urbanicity and unemployment rate, adjusted for age and marital status

^c^Model 3: includes individual SES, urbanicity and environmental pollution, adjusted for age and marital status

^d^Model 4: includes individual SES, urbanicity and unemployment rate and industrial pollution, adjusted for age and marital status

**Table 3:** Age-adjusted lung cancer mortality rate ratios (MRR) with 95% confidence intervals (CI) by individual and sub-district characteristics 2001–2011, women aged 65+.

	Model 1^a^		Model 2^b^		Model 3^c^		Model 4^d^	
	MRR	95%CI	MRR	95%CI	MRR	95%CI	MRR	95%CI
**Individual characteristics**								
*Educational level*								
Pre-primary/primary (ref.)	1.00	-	1.00	-	1.00	-	1.00	-
Lower secondary	0.93	(0.87–1.00)	0.93	(0.87–1.00)	0.93	(0.87–1.00)	0.93	(0.87–0.99)
Upper and post secondary	0.97	(0.89–1.06)	0.97	(0.89–1.05)	0.97	(0.89–1.05)	0.96	(0.88–1.05)
Tertiary	0.82	(0.73–0.92)	0.81	(0.72–0.91)	0.81	(0.72–0.91)	0.81	(0.72–0.91)
*Comfort level*								
Low quality (ref.)	1.00	-	1.00	-	1.00	-	1.00	-
Basic quality	0.94	(0.85–1.03)	0.94	(0.86–1.03)	0.94	(0.85–1.03)	0.94	(0.86–1.03)
Good quality	0.92	(0.83–1.01)	0.92	(0.84–1.02)	0.92	(0.83–1.01)	0.92	(0.84–1.02)
High quality	1.00	(0.90–1.11)	1.00	(0.90–1.11)	1.00	(0.90–1.11)	1.00	(0.90–1.11)
Very high quality	0.94	(0.82–1.07)	0.94	(0.83–1.07)	0.94	(0.82–1.07)	0.94	(0.83–1.07)
*Home ownership*								
Tenant (ref.)	1.00	-	1.00	-	1.00	-	1.00	-
Owner	0.65	(0.61–0.69)	0.65	(0.61–0.69)	0.65	(0.61–0.69)	0.65	(0.61–0.69)
*Marital status*								
Non-married (ref.)	1.00	-	1.00	-	1.00	-	1.00	-
Married	0.78	(0.73–0.83)	0.78	(0.74–0.83)	0.78	(0.73–0.83)	0.78	(0.74–0.83)
**Sub-district characteristics**								
*Urbanicity*								
Non-urban (ref.)			1.00	-	1.00	-	1.00	-
Urban			1.30	(1.16–1.46)	1.33	(1.20–1.48)	1.30	(1.15–1.47)
*Unemployment rate*								
Q1 (lowest) (ref.)			1.00	-			1.00	-
Q2			1.04	(0.90–1.21)			1.03	(0.88–1.20)
Q3			1.30	(1.13–1.49)			1.29	(1.08–1.54)
Q4 (highest)			1.16	(0.99–1.34)			1.14	(0.94–1.39)
*% Employed in mining*								
Q1 (lowest) (ref.)					1.00	-	1.00	-
Q2					1.07	(0.93–1.23)	1.06	(0.93–1.21)
Q3					1.09	(0.95–1.26)	1.00	(0.85–1.17)
Q4 (highest)					1.22	(1.06–1.41)	1.03	(0.86–1.23)
*% Employed in other high-risk industries*								
Q1 (lowest) (ref.)					1.00	-	1.00	-
Q2					0.84	(0.72–0.97)	0.87	(0.76–1.00)
Q3					0.96	(0.83–1.11)	0.98	(0.86–1.12)
Q4 (highest)					0.99	(0.86–1.13)	1.05	(0.92–1.20)

Ref. = Reference group

^a^Model 1: includes individual SES, adjusted for age and marital status

^b^Model 2: includes individual SES, urbanicity and unemployment rate, adjusted for age and marital status

^c^Model 3: includes individual SES, urbanicity and environmental pollution, adjusted for age and marital status

^d^Model 4: includes individual SES, urbanicity and unemployment rate and industrial pollution, adjusted for age and marital status

Controlling for individual SES results in a 15.7% decrease of the sub-district-level variation compared to the null model among women (Table 1; model 1). Regional variation in individual SES thus accounts for a small part of the sub-district variation in female lung cancer mortality. Among men, the inclusion of individual SES results in a change of 6.9% in regional variation.

### Association of sub-district-level characteristics with lung cancer mortality

When the sub-district characteristics are included (model 4), the effects of individual SES remain unchanged, indicating that there is little confounding between the individual and sub-district level variables included in the model. In line with the spatial pattern observed in the map (Fig 2), lung cancer mortality among women in urban areas is substantially higher compared to non-urban areas (MRR = 1.30 [95%CI = 1.16–1.46]) (model 2). Among men there are few differences in lung cancer mortality by urbanicity.

Sub-districts with a higher unemployment rate appear to have increased levels of lung cancer mortality among both men and women, when controlled for individual SES and urbanicity (model 2). Especially residents living in sub-districts in the 3th quartile of unemployment experience higher lung cancer mortality risks (MRR_men Q3_ = 1.13 [95% CI = 1.05–1.23]; MRR_women Q3_ = 1.30 [95%CI = 1.13–1.49]). For the sub-district proportion working in high-risk industries (model 3), the association with lung cancer mortality depends on the type of industry. The percentage working in mining in the sub-district is associated with higher lung cancer mortality, especially among men. Female lung cancer mortality is higher among women living in sub-districts with the highest proportion of miners (MRR = 1.22 [95%CI = 1.06–1.41]). Other high-risk industries do not appear to be associated with increased lung cancer mortality. The addition of all sub-district characteristics in the full model (model 4) results in contrasting changes in the effects of unemployment rate for men and industry for women. Among women, lung cancer mortality in mining areas is no longer significantly elevated after unemployment rate is included (model 3 vs. 4). The effects of urbanicity, unemployment rate and other high-risk industries on female lung cancer mortality remain unchanged. For men, the effect of unemployment is largely attenuated by the inclusion of the industry variables and a reverse pattern can now be observed, with lower mortality rates among sub-districts with a high unemployment rate (model 2 vs. 4). The elevated levels of male lung cancer mortality observed for mining remain in all quartiles (model 3 vs. 4). Men living in sub-districts with the highest percentage of miners experience up to 30% higher lung cancer mortality (MRR = 1.30 [95%CI = 1.18–1.43]) compared to men in sub-districts with the lowest percentage of miners (model 4). When going more in-depth into the type of mining, a higher lung cancer mortality risk is mainly observed in sub-districts with a high percentage of mines other than coal mining (results not shown).

The sub-district variation in lung cancer mortality decreases substantially after including the sub-district characteristics in the model (Table 1, model 2 and 3). After including all sub-district characteristics in addition to individual SES (model 4), the ARD is reduced by 41% among men and 63% among women compared to the regional variation observed in the null model. Sub-district characteristics thus explain part of the geographical variation in lung cancer mortality in both men and women.

## Discussion

The aim of this study was to map out sub-district-level variation in lung cancer mortality in Belgium, and to examine to what extent individual and area SES and industrial pollution contribute to the observed geographical patterns. This is one of the first studies on lung cancer mortality investigating the combined effect of individual and area level SES and industrial exposure. The results show clear regional disparities in lung cancer mortality in Belgium but also disparities in the geographical pattern for men and women aged 65+. While lung cancer mortality among men is mainly elevated in the east of Belgium, women experience higher lung cancer mortality rates in urban areas.

In line with previous studies [7,8], an association between individual SES and lung cancer mortality is found. Individuals who are highly educated, living in owner-occupied and high-quality housing have a lower risk of lung cancer mortality. However, individual characteristics do not appear to be a major contributor to regional lung cancer mortality differences. Sub-district characteristics have a modest effect on lung cancer mortality. This finding is consistent with previous research results showing an association between area-characteristics on lung cancer mortality, independently of individual SES; the most deprived areas having a higher lung cancer mortality compared to the least deprived [14,16–18]. Controlling for smoking, occupational and environmental exposure in these studies could not fully explain elevated lung cancer mortality risks in deprived areas [17]. Area deprivation might impact health and mortality through social norms on health behavior, lack of social organization and support, limited access to healthcare and other services, or through an unhealthy physical environment [12,37]. Not all studies did find an effect of area deprivation however [15]. Studies including the proportion of manual workers, social cohesion [19] and median income [38] for instance did not report a significant association with lung cancer mortality.

The outcomes of this study, on the other hand, show that area characteristics should not be overlooked, even though they seem to be less associated with lung cancer mortality, compared to individual-level characteristics. Area deprivation, measured by the sub-district-level unemployment rate, is associated with increased lung cancer mortality. After controlling for industrial exposure, this association is largely attenuated for men but not for women, suggesting a larger impact of area deprivation for women. Stafford et al. (2005) also found a stronger impact of neighborhood characteristics on women’s health, possibly because women tend to spend more time in the neighborhood they live in or simply because they are more vulnerable to neighborhood characteristics [39].

Urbanicity appears to be another determinant of female lung cancer mortality. Women in urban areas have a 30% higher risk of dying from lung cancer than women living in non-urban areas, even when controlling for individual SES, area deprivation and industrial exposure. Among men, differences between urban and non-urban sub-districts were not observed. Elevated lung cancer risks in urban areas were observed in other European studies as well, albeit for both sexes [40,41]. Area deprivation could explain only part of the urban excess in lung cancer mortality in these studies [41]. A higher smoking prevalence among women in urban areas has been observed in several Western-European countries [42]. Smoking behavior thus might explain the observed association between urbanicity and female lung cancer mortality.

While the unemployment rate and urbanicity showed little associations to male lung cancer mortality, industry does appears to have an association. Men living in sub-districts with mining industry have higher lung cancer mortality, with up to 30% increased risks in the sub-districts with the highest proportion of miners. Especially mining types other than coal mining seem to cause this effect. A possible explanation might be environmental pollution, as mining dust and aerosol particles include several carcinogenetic substances [32], and elevated metal concentrations were found near mining operations [43]. However, the results do not indicate substantial increased lung cancer mortality among women living in the same sub-districts. Possibly men living in mining sub-districts have a higher risk of occupational exposure by being employed in these types of mining. A study on lung cancer incidence among miners found a higher lung cancer risk among men employed in mining, especially ore miners and quarrymen. The association remained significant after controlling for smoking and occupational exposure from working in other at-risk occupations [44]. To incorporate possible occupational exposure, the analysis was repeated for a subset of the male study population including individual occupation in high-risk industries in 1981. The inclusion of individual occupation slightly reduces the mortality rates in areas with a high percentage employed in mining, yet mining areas continue to show elevated levels of male lung cancer mortality (results not shown). Unmeasured individual occupational exposure before 1981, other unmeasured area characteristics, or actual environmental pollution might underlie this elevated risk.

### Strengths and limitations

One of the strengths of this study is the use of a unique dataset covering the total Belgian population with mortality information from 2001 to 2011, including all lung cancer deaths during this period. The data include extensive information on socioeconomic and demographic variables, which makes it possible to measure several aspects of individual SES including education, housing comfort and home ownership. Each of these variables measures a different aspect of SES which is necessary to capture the complete picture of socioeconomic status [28]. As multiple indicators are used to measure individual SES, the risk that the observed effects of sub-district-level characteristics are due to unmeasured individual characteristics is reduced [13,34]. When data on individual SES are unavailable, studies often rely on aggregated area-level data to make assumptions on individuals, risking the so-called ecological fallacy [45]. By including both individual and area-level measures, this has been avoided in this study. This study also looked into the combined effect of area deprivation and industrial pollution, and as the results show, it might be important to incorporate both factors, as the sub-district’s unemployment level and the percentage employed in mining and in other high-risk industries mitigate each other’s effect.

The use of multilevel analysis enables us to study the variation in lung cancer mortality, and the effects of SES and industrial exposure, both between individuals and between sub-districts. Additionally, it takes into account the hierarchical structure of the data in which individuals are nested within sub-districts, producing more reliable outcomes [34]. A random intercept model is used, allowing lung cancer mortality to vary at sub-district level. It is unlikely that the associations between individual and sub-district level characteristics differ across sub-districts, which is why a random slope model (allowing these factors to vary across sub-districts) was not used.

Inherent to multilevel studies into the effects of area-characteristics on health and mortality, there are certain methodological considerations, including the geographical unit to be used, the choice of area-level variables and the possibility of confounding factors [34,45]. The optimum geographical level to study area-effects on health and mortality is much debated [46,47]. Although area characteristics of both local neighborhoods and larger geographical units appear to affect mortality, the largest effects are generally observed for small-scale areas [13]. By using sub-district level, we ensured that the different regions contain enough cases to maintain stable results, and are detailed enough to distinguish between urban and non-urban areas. Analysis at smaller geographical levels would likely produce more unstable and unreliable results due to the small number of lung cancer deaths by area. To test the robustness of the area effects at sub-district level, the models were repeated at municipal level. This did not produce substantially different outcomes, except for a stronger effect of unemployment rate, especially in women (results not shown). Duration of residence in an area and selective migration might influence the effect of area-characteristics as well [12]. This is unlikely to bias our results as more than 90% of the study population had a stable residence for over 10 years (based on their municipality of residence in 1991 and 2001). The models produced similar outcomes when including the individuals with a long-term residence only (results not shown).

The sub-district level unemployment rate and the percentage employed in mining and in other high-risk industries are constructed from aggregated individual census data. They are therefore so-called derived variables, in contrary to integral variables, which are area characteristics without an equivalent at individual level that can be measured only at area-level [45]. However, the total population is used to calculate the sub-district’s unemployment rate and employment in mining and other high-risk industries, minimizing overlap between individual and area-level variables within the study population. Although the full model including individual and sub-district-level SES and high-risk industry explains a large part of regional variation in lung cancer mortality (63% for women and 41% for men), significant regional variation remains. This suggests an impact of unmeasured factors.

A possible underlying determinant of the geographical pattern in lung cancer mortality is smoking behavior. Smoking is an important risk factor for lung cancer mortality, as 90% of lung cancer deaths in European men and 60% of lung cancer deaths in European women are expected to be tobacco-related [48]. Belgium not only has one of the highest male lung cancer mortality rates in Europe, but also used to have one of the highest per capita consumption of tobacco [49]. In the 1950s, an estimated 77% of Belgian men were smoking, this decreased to 53% in 1980 and further declined to 34% in 2001 [50]. The share of smokers among Belgian women remained stable over time: 20% in 1955, 21% in 1980, and 24% in 2001 [50]. These smoking trends result in strong cohort effects in lung cancer mortality rates among Belgian men [48]. The Belgian census does not include information on lifestyle, so it was not possible to control for smoking. Data from the Belgian Health Interview Survey (HIS) can give some insight into smoking behavior, although this survey is not as extensive as the population-based data used in this study and can only be disaggregated to the less-detailed provincial level (NUTS2). Results from the HIS from 2001 show no differences in the share of ever smokers by educational level. However, current smoking is less common among higher educated individuals (22%), compared to lower educated persons (30%). Heavy smoking is also less common among high educated individuals, although they do have a higher share of occasional smokers [51]. The higher smoking prevalence and higher share of heavy smokers among low educated individuals might contribute to the higher lung cancer mortality risk we observe in this group. On the other hand, the results of a Dutch study show that neither smoking nor occupational exposure could account for higher lung cancer incidence rates among low-educated individuals [8,52]. Compared to men with primary education only, the MRR of high educated men changed from 0.50 (95% CI 0.35–0.75) to 0.52 (95% CI 0.39–0.82) after controlling for smoking behavior and intake of β-carotene, vitamin C and retinol [8]. Additionally adjusting for occupational exposure to carcinogens resulted in a MRR of 0.53 (95% CI 0.34–0.82) for high educated men [52].

Smoking might influence the geographic pattern in lung cancer mortality as well. Regional differences in smoking have been found to contribute to regional variation in all-cause mortality [53] and lung cancer specific mortality [54] in the Netherlands. Also in Belgium, the geographical pattern for lung cancer incidence and tobacco prevalence (measured by the incidence of COPD) appear to overlap [6]. Data from the HIS from 2001, 2004 and 2008 were used to get an indication of the geographical pattern of smoking for survey participants aged 65+. Smoking behavior among men aged 65+ did not significantly differ across provinces, with the exception of ever smokers. Especially Limburg, a province in the northeast, showed a high proportion of ever smokers, which might contribute to higher male lung cancer mortality in this region. Differences in smoking behavior could be an explanation for the significantly higher lung cancer mortality among women living in urban areas, which persisted even after controlling for individual SES and other area characteristics. The Health Interview Surveys (HIS) do not show differences in the proportion ever, current or daily smoking rates among women in urban areas, compared to sub-urban or rural areas. However, these figures are only available for women of all ages, and smoking rates by urbanization level could not be compared for women aged 65+ [55]. Among provinces, significant variations in smoking behavior among women aged 65+ can be observed. Provinces containing sub-districts with high female lung cancer mortality rates have a particularly high share of smokers. However, there are also some exceptions: provinces with a high smoking prevalence but without elevated lung cancer mortality or with a relatively low smoking prevalence but substantially higher levels of lung cancer mortality. Smoking has also been identified as an intermediary variable in the association between area characteristics and lung cancer mortality [17]. Previous studies found an increased smoking prevalence in deprived areas, which could only partly be explained by the individual characteristics of the residents [56]. Other risk factors, such as radon exposure, might contribute to geographical differences in lung cancer mortality as well. According to the Federal Agency of Nuclear Control relatively high radon concentrations are observed in the south of Belgium, with the highest concentrations in the southeast [57]. Radon could partially contribute to lung cancer mortality in these areas with high radon concentrations. However, sub-districts with the highest levels of lung cancer mortality show little overlap with areas with high radon concentrations. Other environmental risk-factors such as air pollution might have a possible influence on regional variation in lung cancer mortality as well [25].

Future studies should consider including additional variables to gain a better understanding of the effects of area characteristics on regional variation in lung cancer mortality. Controlling for smoking and long-term occupational exposure at the individual level is likely to result in more precise estimates of the association between sub-district characteristics and lung cancer mortality. In addition, more precise measurements of industrial pollution might result in stronger associations with lung cancer mortality. Data on concentrations of pollutants in the soil and air (from both industry and other sources such as traffic) might allow for a better insight into the association between lung cancer mortality and pollution at the sub-district level and are thus highly relevant for future study.

Yet, this study contributes to the limited knowledge on the association between lung cancer mortality and both individual and area-level characteristics simultaneously. The results indicate that there are substantial differences in lung cancer mortality by individual SES. Targeted anti-smoking measures specifically addressing low-SES individuals could ensure further declines in lung cancer among Belgian men and prevent a further increase among women in the future. Our study also gave insight into geographic patterns in lung cancer mortality, and pointed to areas with significantly higher levels of lung cancer mortality. Studies into regional mortality differences are mentioned as helpful tools in discovering lung cancer causes other than tobacco consumption [54]. The results of our study point to a possible association between high-risk industries, especially mining, and elevated lung cancer risks.

## Conclusion

This study found a strong association between individual SES and lung cancer mortality. Individuals with a low SES experience significantly higher levels of lung cancer mortality. Sub-district level characteristics appear to have an influence on lung cancer mortality as well: in women an association between unemployment rate and lung cancer mortality was observed; in men an association between the percentage employed in mining and lung cancer mortality was observed. In addition to variation in lung cancer mortality between individuals, significant regional variation in lung cancer mortality between sub-districts in Belgium was found. Individual SES could only partly explain this regional variation. The bulk of lung cancer mortality’s sub-district variation could be explained by sub-districts unemployment rate, the percentage employed in mining and the percentage employed in other high-risk industries, suggesting that both individual- and area-level characteristics are important contributors to regional differences in lung cancer mortality.
